# Androstenedione and testosterone but not progesterone are potential biomarkers of pregnancy in Humpback Whales (*Megaptera novaeangliae*) approaching parturition

**DOI:** 10.1038/s41598-020-58933-4

**Published:** 2020-02-19

**Authors:** Greta Dalle Luche, Ashley S. P. Boggs, John R. Kucklick, Jasmin Groß, Darryl W. Hawker, Susan Bengtson Nash

**Affiliations:** 1 Environmental Futures Research Institute, Griffith University, Brisbane, QLD 4111 Australia; 2 National Institute of Standards and Technology, Hollings Marine Laboratory, Charleston, SC 29412 USA; 3 School of Environment and Science, Griffith University, Brisbane, QLD 4111 Australia

**Keywords:** Reproductive biology, Endocrinology, Conservation biology, Population dynamics

## Abstract

The blubber steroid hormone profiles of 52 female humpback whales migrating along the east coast of Australia were investigated for seasonal endocrine changes associated with reproduction. Individuals were randomly sampled during two stages of the annual migration: before reaching the breeding grounds (northward migration; June/July), and after departing from the breeding grounds (southward migration; September/October). Assignment of reproductive status of the sampled individuals was based on season, single-hormone ranks and multi-variate analysis of the hormonal profiles. High concentrations of progesterone (>19 ng/g, wet weight), recognised as an indicator of pregnancy in this species, were only detected in one sample. However, the androgens, testosterone and androstenedione were measured in unusually high concentrations (1.6–12 and 7.8–40 ng/g wet weight, respectively) in 36% of the females approaching the breeding grounds. The absence of a strong accompanying progesterone signal in these animals raises the possibility of progesterone withdrawal prior to parturition. As seen with other cetacean species, testosterone and androstenedione could be markers of near-term pregnancy in humpback whales. Confirmation of these androgens as alternate biomarkers of near-term pregnancy would carry implications for improved monitoring of the annual fecundity of humpback whales via non-lethal and minimally invasive methods.

## Introduction

Steroid hormones are the principal mediators of reproduction and stress response in mammals. As such, they have been increasingly used as health biomarkers in terrestrial and aquatic wildlife, including cetaceans^1^. In cetaceans, endogenous steroids have been measured both in traditional (blood, saliva) and in remotely accessible biological samples (blubber biopsies, faeces, and respiratory vapour/blow^2–4^) which have extended the application of these biomarkers to free-swimming individuals with minimal disturbance to them. Diagnostic hormonal techniques based on remote sampling allow monitoring of fecundity^5^ and stress exposure^6^ in wild cetacean populations. This is particularly relevant for large, filter-feeding (baleen) whales for which longitudinal sampling of individuals in the wild is logistically challenging^7^.

Use of hormonal biomarkers in the absence of life history information requires comprehensive understanding of species-specific endocrinology^8^. However, comparatively few endocrine investigations have been undertaken on humpback whales (HW; *Megaptera novaeangliae*). Lack of hormonal baselines can result in suboptimal or misleading interpretation of steroid hormone biomarkers in this species. Previous investigations of HW endocrinology in free-swimming individuals have primarily focused on the determination of female reproductive status for pregnancy rate calculation, by measurement of the single steroid hormone, progesterone^9–12^. Progesterone is a fundamental hormone that establishes and sustains  pregnancy in the majority of mammals^13^. Circulating progesterone in pregnant females is generally higher than in non-pregnant females by orders of magnitude^14^. Such acute differences in concentration have enabled blubber progesterone to be validated as a biomarker of pregnancy in various cetacean species^2,15^, including HWs^12^. Traditionally, the non-invasive verification of pregnancy in large whales has required the collection of life history information through calf sightings^16,17^. Pallin *et al*.^12^ combined this traditional approach with blubber progesterone analysis by biopsying female HWs two to nine months after the peak of the breeding season. This study showed that female HWs with blubber progesterone concentrations >54 ng/g (wet weight, ww) were accompanied by a calf the following year. These data were then used to build a model assessing pregnancy probability on the basis of blubber progesterone concentration alone. Pallin *et al*. ’s study^12^ represents the most advanced method validation presented to date, although it still did not provide any information on progesterone concentrations during the final three months of HW gestation.

For samples collected outside of the validated gestation interval, the same progesterone concentration threshold is not guaranteed to produce reliable pregnancy predictions. In some cetacean species, such as bottlenose dolphins and killer whales, circulating progesterone concentrations have been shown to decrease through pregnancy^18–20^. It cannot be excluded that a similar mechanism might occur in HWs. By using pregnancy assignment through progesterone concentration, Clark *et al*.^9^ measured pregnancy rates in HWs through two consecutive sampling periods within the same year: three to four and eight to nine months after the peak of conceptions, respectively. Across this period, observed pregnancy rates dropped from 64% to 11%, with the lower pregnancy rates observed among females at a more advanced stage of gestation. Shifting the time of sampling for steroid hormone analysis may affect the pregnancy rate calculations in multiple ways. For example, shifting the sampling towards the end of the gestation period inherently excludes pregnancies terminated early. However, in the absence of comprehensive longitudinal observations of progesterone in HWs, it is possible that assay of this hormone alone might not be sufficient to accurately determine pregnancy.

Due to uncertainties surrounding progesterone concentrations across  the full gestation period, other steroid hormones involved in reproduction may provide an additional dimension to the evaluation of an individual’s reproductive status. For example, androgens have also been shown to increase during cetacean pregnancy, as observed in individuals of both toothed^5,21–23^ and baleen whales^4^. Studies that allowed longitudinal monitoring of testosterone and androstenedione in pregnant individuals observed a monotonic temporal increase in these hormones through three quarters of the gestation period. Multiple steroid hormone analysis could augment and provide a more robust indicator of reproductive status compared to progesterone-only assays.

Recently, Dalle Luche *et al*.^24^ validated a liquid chromatography-tandem mass spectrometry (LC-MS/MS) method for the simultaneous analysis of 11 steroid hormones in HW blubber. Measuring a suite of steroid hormones from a single blubber sample, instead of a single hormone, can provide more specific information on the physiology of an individual^25^. Multiple hormones in concert control the majority of physiological processes. Therefore, by considering a suite of steroid hormones, interpretation and assignment of a given endocrine status is more thorough than measuring individual hormone signals^4,25^.

In addition to allowing the simultaneous measurement of multiple steroid hormones, the use of LC-MS/MS also carries some technical advantages over the generally used immunoassays. By directly measuring the target steroid hormone on the basis of its physico-chemical properties, rather than its biological reactivity with an antibody, LC-MS/MS enables higher accuracy and selectivity among certain classes of metabolites that may share similar reactivity. The application of a LC-MS/MS multi-hormone method on blubber collected from free-roaming HWs during the breeding season has the potential to provide new insights into the endocrinology of this species.

In this study we simultaneously analysed 11 steroid hormones in blubber biopsies collected from female Southern Hemisphere HWs travelling along the East Australian migration corridor at two phases of their annual migration viz. to and from the breeding grounds. In HWs, breeding is known to occur during the migration, with birth expected upon arrival at the calving grounds the following year^26^. Non-pregnant, ovulating, early-pregnant and near-term pregnant females are known to co-occur during the migration. By targeting individual females at two time points during the breeding season, we provide novel insight into female HW endocrinology at various reproductive stages during a season, which has not been addressed by previous studies. We investigate the general and seasonal structures of the obtained endocrine profiles of each whale, the relationship between steroid content and sample lipid percentage, and propose reproductive categories based on past steroid hormone research conducted on HWs as well as other related species.

## Results

### Steroid profiles

The blubber steroid profiles obtained from the analysis of 52 female humpback whales are expressed herein as ng/g ww (Table 1). Steroid detection frequency was not homogenous in the sample set. Progesterone was quantified in almost all of the samples (94%), followed by androstenedione (82%), cortisol (72%), and testosterone (53%). By contrast, some steroid hormones were only quantifiable in a relatively limited number of samples (e.g. oestrone, 27%; 17-hydroxyprogesterone, 23%; and 11-deoxycorticosterone, 23%). The remaining compounds investigated (i.e. cortisone, corticosterone, 11-deoxycortisol and oestradiol) could not be quantified in any of the biopsies and are therefore not discussed further.

**Table 1:** Sample information and steroid profiles (ng/g, ww).

Sample ID	Season	Sighting information	17-Hydroxy-progesterone	Testosterone	Androstenedione	Progesterone	Cortisol	11-Deoxy-corticosterone	Oestrone
N16.01	NM		<0.26	0.21	0.97	0.42	2.1	<0.64	<0.17
N16.04	NM		<0.26	0.15	0.87	0.11	0.71	<0.64	<0.17
N16.07	NM		<0.26	<0.15	<0.028	0.15	0.67	<0.64	<0.17
N16.15	NM		<0.26	0.19	1.8	0.25	<0.24	2.0	<0.17
N16.17	NM		<0.26	<0.15	0.02	0.10	0.27	<0.64	<0.17
N16.19	NM		<0.26	2.4	7.8	0.11	0.25	<0.64	0.44
N16.21	NM		<0.26	<0.15	<0.028	<0.065	<0.24	<0.64	<0.17
N16.22	NM		<0.26	0.25	1.7	0.38	0.4	1.6	<0.17
N16.23	NM		0.93	4.3	8.6	0.33	0.82	0.38	0.17
N16.28	NM		<0.26	<0.15	0.030	0.15	0.37	<0.64	<0.17
N16.32	NM		<0.26	<0.15	0.030	0.11	0.87	<0.64	<0.17
N16.33	NM		<0.26	<0.15	<0.028	0.11	1.0	<0.64	<0.17
N16.37	NM		0.43	5.9	24	0.23	0.87	0.66	0.45
N16.48	NM		<0.26	<0.15	<0.028	<0.065	0.34	<0.64	<0.17
N16.52	NM		<0.26	<0.15	<0.028	0.10	0.27	<0.64	0.15
N16.53	NM		1.6	11.9	17	0.64	1.3	1.6	0.59
N16.58	NM		1.7	8.8	32	1.0	1.3	1.6	0.76
N16.61	NM		2.2	11	11	1.3	0.86	2.3	0.38
N17.20	NM		<0.26	<0.15	<0.028	<0.065	0.56	2.3	0.60
N17.25	NM		<0.26	<0.15	0.060	0.070	<0.24	<0.64	<0.17
N17.29	NM		<0.26	<0.15	0.050	0.090	<0.24	<0.64	<0.17
N17.31	NM		1.0	8.1	40	0.32	<0.24	5.5	0.45
S15.04	NM		1.1	1.6	24	0.48	0.58	7.4	<0.17
S15.08	SM		0.49	1.59	5.6	0.49	0.45	<0.64	0.65
S15.11	SM		<0.26	2.29	3.7	0.25	<0.24	<0.64	0.13
S15.12	SM		0.45	0.68	3.2	0.21	<0.24	<0.64	0.25
S15.19	SM		<0.26	<0.15	0.040	2.6	0.24	0.74	<0.17
S15.22	SM		<0.26	0.6	2.9	0.07	<0.24	0.31	<0.17
S15.24	SM	Recent mother	<0.26	0.22	0.070	0.19	0.28	<0.64	<0.17
S15.27	SM		<0.26	<0.15	0.050	0.07	0.63	<0.64	0.63
S15.30	SM		<0.26	0.83	3.2	0.12	<0.24	<0.64	<0.17
S15.31	SM		<0.26	<0.15	<0.028	6.2	0.24	<0.64	<0.17
S15.32	SM		<0.26	0.35	0.050	2.0	<0.24	<0.64	<0.17
S15.35a	SM	Recent mother	0.88	0.27	0.030	22	0.27	0.44	<0.17
S15.35b	SM	Recent mother	<0.26	0.25	0.040	0.13	0.4	0.25	<0.17
S15.37	SM	Recent mother	<0.26	<0.15	0.030	0.15	<0.24	<0.64	0.21
S15.50	SM	Recent mother	<0.26	0.21	0.030	1.5	0.32	0.25	<0.17
S15.51	SM		<0.26	0.94	2.74	0.2	<0.24	<0.64	<0.17
S15.54	SM		0.85	<0.15	0.030	8.8	0.26	0.35	<0.17
S15.55	SM	Calf-of-the-year	<0.26	0.29	0.030	<0.065	0.38	<0.64	<0.17
S15.59	SM		<0.26	0.17	1.0	0.08	<0.24	0.33	<0.17
S15.62	SM	Recent mother	<0.26	0.18	0.030	0.18	0.25	<0.64	0.12
S15.64	SM		<0.26	2.0	6.4	0.25	<0.24	<0.64	<0.17
S15.67	SM	Recent mother	<0.26	<0.15	0.050	0.11	0.31	<0.64	0.12
S16.02	SM		NA	<0.15	0.030	0.65	0.35	<0.64	<0.17
S16.06	SM		NA	<0.15	<0.028	0.13	0.87	<0.64	<0.17
S16.12	SM		NA	<0.15	<0.028	4.0	1.0	<0.64	<0.17
S16.15	SM		NA	<0.15	0.030	12	0.63	<0.64	<0.17
S17.03	SM		<0.26	<0.15	0.060	17	1.1	<0.64	<0.17
S17.08	SM	Recent mother	<0.26	<0.15	0.040	0.16	0.85	0.66	0.19
S17.11_	SM		<0.26	<0.15	<0.028	13	1.5	<0.64	0.23
S17.12	SM	Recent mother	<0.26	<0.15	<0.028	10	0.94	<0.64	0.20

Notes: “NA” indicates that the concentration could not be determined due to contingent reasons. Samples are classified on the basis of their time of sampling, or swimming direction, as “northward migration” (NM) or “southward migration” (SM). Individuals were classified as ‘recent mother’ if they were observed travelling alone with an accompanying calf.

### Correlation analysis

Androstenedione and testosterone were strongly positively correlated when migration time points, NM (r_s_ = 0.930, p < 0.001) and SM (r_s_ = 0.868, p < 0.001), were considered separately, as well as when the entire cohort was considered. Cortisol was found to be moderately positively correlated with lipid percentage (r_s_ = 0.259, p = 0.043), although this correlation did not persist when the two sample cohorts were divided.

### Single-hormone and lipid percentage inter-seasonal data differences

Progesterone concentrations were significantly different (χ2 = 4.89, p < 0.027) between NM and SM samples (median_NM_ = 0.148 ng/g; median _SM_ = 0.245 ng/g). As expected, and as previously shown^27^, lipid percentages were also higher (median_NM_ = 57.2%, median_SM_ = 45.7%) in the NM samples (χ2 = 10.3, p = 0.001). Despite the low percentage of detection, 11-deoxycorticosterone also resulted in significantly higher levels in the NM samples (χ2 = 8.72, p < 0.003). The remaining steroid hormones did not show any significant differences in median levels between NM and SM samples.

### Concentration patterns and ranks

**Figure 1:**
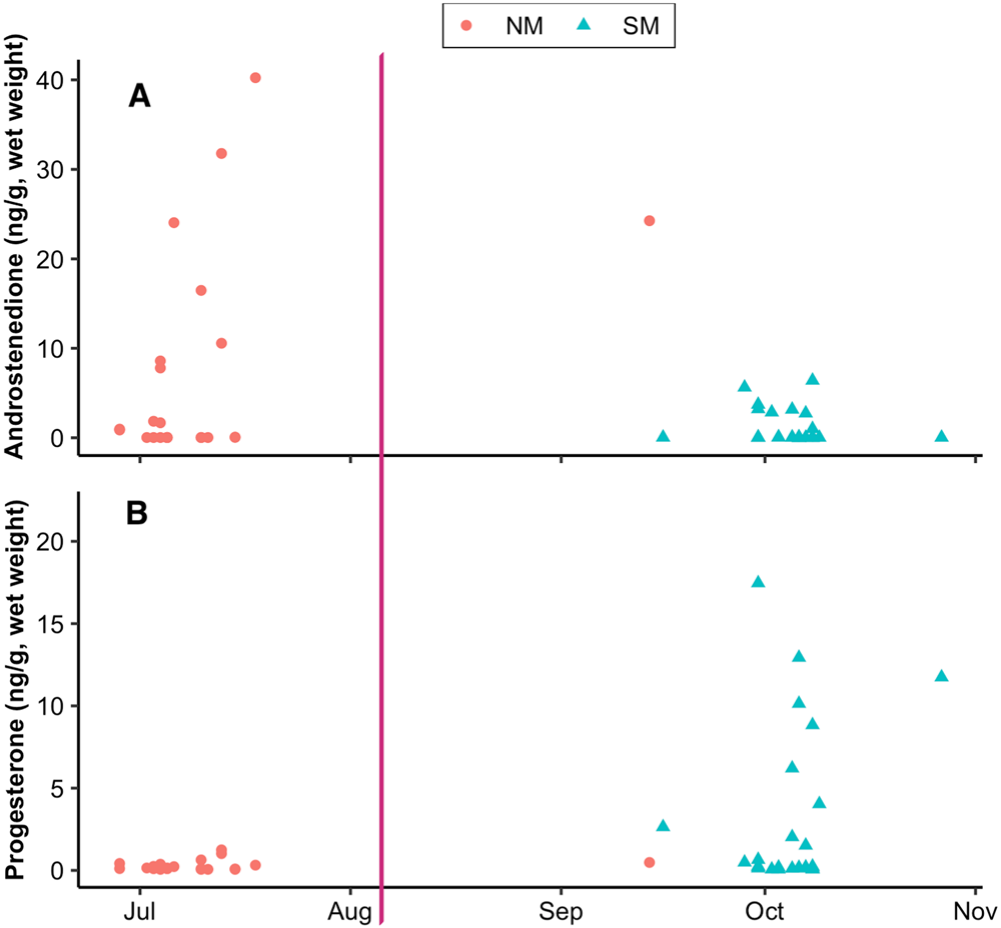
Blubber androstenedione (**A**) and progesterone (**B**) concentrations (ng/g, ww) of female Southern Hemisphere humpback whales, plotted according to the sampling day and migration stream (“Northward Migration”, NM, ; “Southward Migration”, SM, ). The vertical purple line represents the estimated time for peak frequency of conceptions and births in this species (Chittleborough, 1958).

Despite the lack of an overall seasonal trend, single hormone concentrations showed wide ranges with gaps in these ranges among individuals sampled during the same season. Figure 1 shows how androstenedione and progesterone concentrations were distributed among samples across the migration. For the NM samples, androstenedione concentrations spanned from below the RL (<0.028 ng/g) to a maximum concentration of 40 ng/g, with two gaps in concentrations between 0.27 ng/g and 0.87 ng/g, and between 1.8 ng/g and 7.8 ng/g. Clustering of androstenedione concentrations in the SM samples exhibited a gap in concentration between 0.070 ng/g and 1.0 ng/g, similar to the gap between 0.27 ng/g to 0.87 ng/g observed among NM samples. Progesterone concentrations ranged between non-reportable (<0.065 ng/g) to a maximum of 22 ng/g. Progesterone levels were typically less than about 1.5 ng/g, and the only ones that were greater all came from the SM data set.

### Intra-seasonal clusters

**Figure 2:**
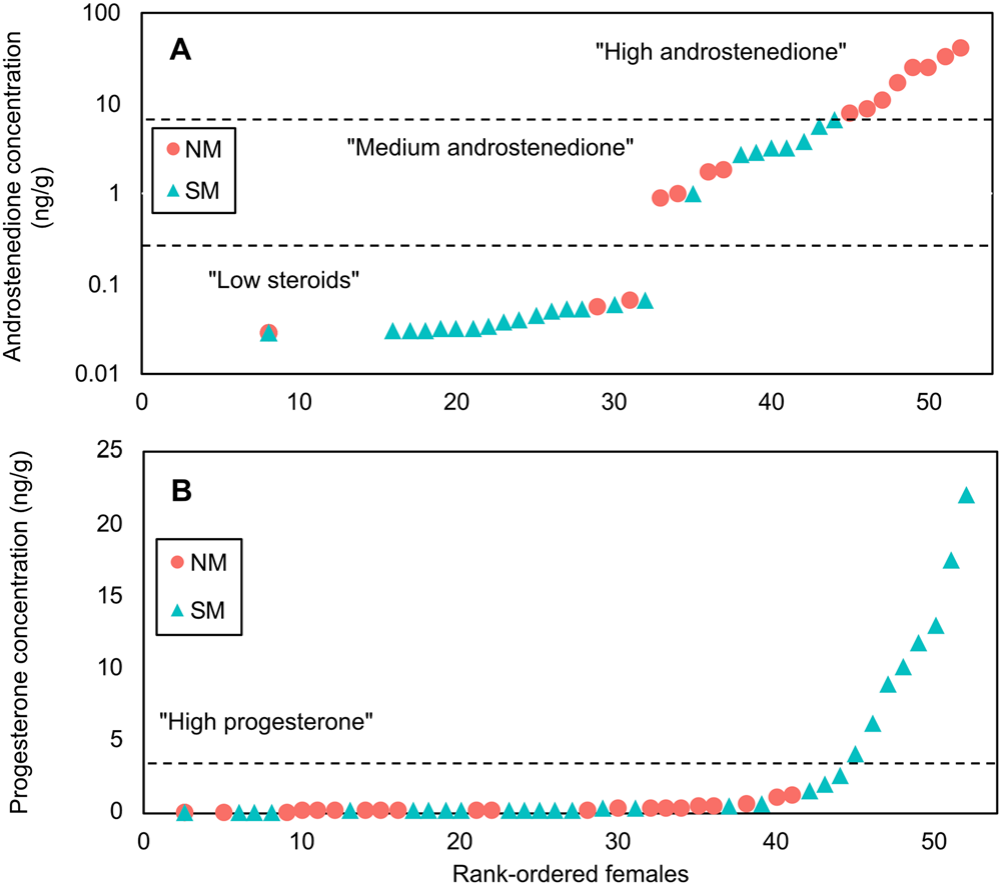
Ranking of blubber androstenedione (**A**) and progesterone concentrations (**B**) in migrating female humpback whales (“Northward Migration”, NM, ; “Southward Migration”, SM, ). Dashed lines denote concentration thresholds that have been chosen as discriminants for sample clustering. Cluster labels are reported in the Figure between quotation marks.

**Figure 3:**
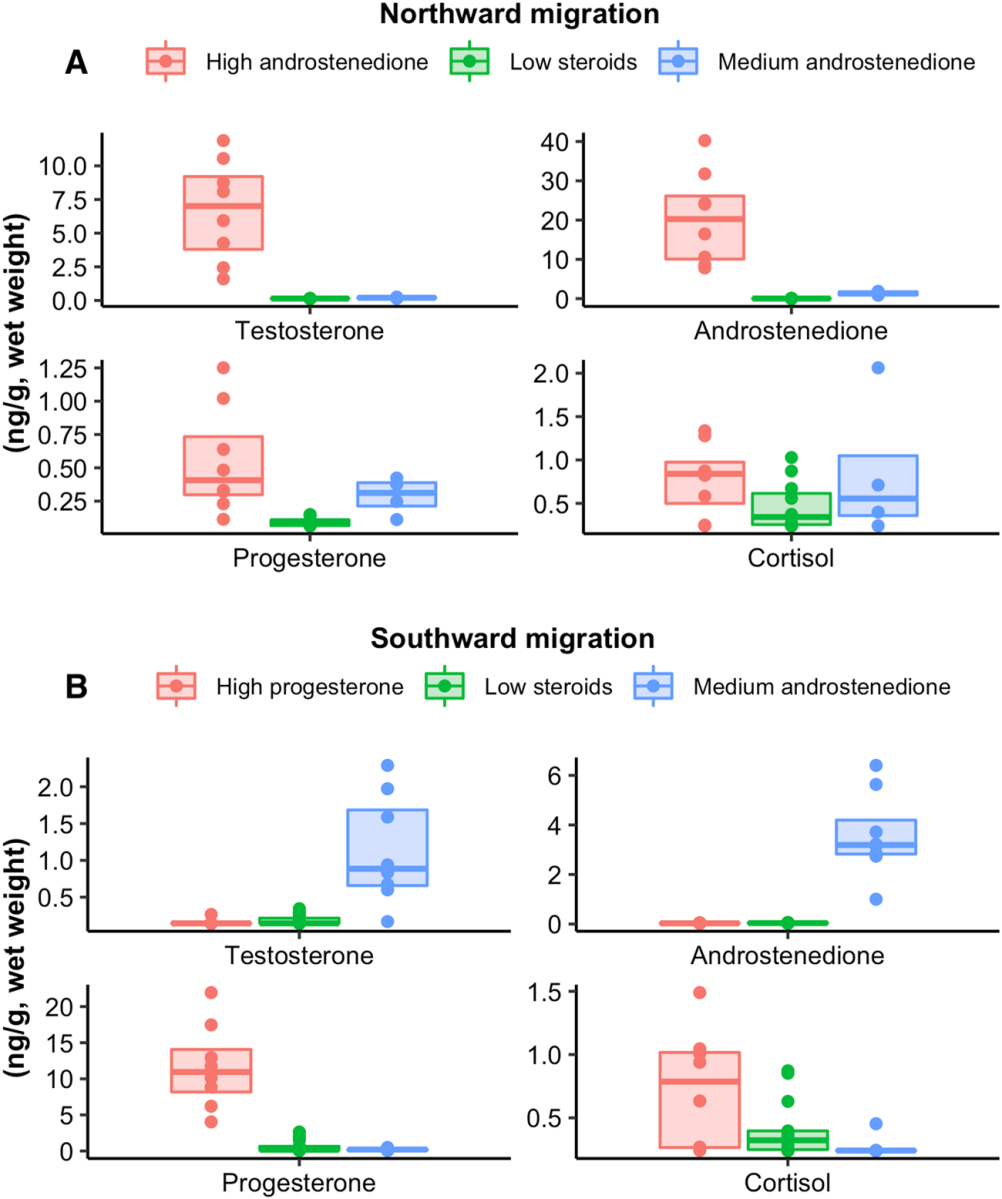
Measured concentrations of testosterone, androstenedione, progesterone, and cortisol (ng/g wet weight) in humpback whale samples. Samples are clustered by season (“Northward Migration”, A or “Southward Migration”, **B**) and steroid hormone profile characteristics. Northward migration samples are classified as “High androstenedione” (androstenedione > 7.8 ng/g), “Medium androstenedione” (0.87 ng/g < androstenedione < 7.8 ng/g) and “Low steroids” (androstenedione < 0.87 ng/g). Southward migration samples are classified as “High progesterone” (progesterone > 4.0 ng/g), “Medium androstenedione” (0.87 ng/g < androstenedione < 7.8 ng/g) and “Low steroids” (androstenedione < 0.87 ng/g). The boxplot centre represents the median value of each hormone, while the lower and upper hinges extend to the first and third quantiles, respectively.

The gaps in hormone distributions identified by sample rankings were used to separate the sample into intra-seasonal clusters (Figs. 2 and 3). Among the NM samples, androstenedione concentrations were used to discriminate between the three concentration clusters found in the data set. Androstenedione was chosen over testosterone as a discriminant biomarker amongst the NM and SM samples for its higher percentage of detection in the samples, and because it showed higher concentrations and wider concentration gaps. Samples featuring androstenedione concentrations greater than or equal to 7.8 ng/g all originated from the NM cohort and were denoted as “High androstenedione”, while those samples with androstenedione concentration below 7.8 ng/g but above 0.87 ng/g, were categorised as “Medium androstenedione”. Samples with progesterone concentration equal to or above 4 ng/g, exclusively present in the SM data set, were separated into a cluster denoted “High progesterone”. Remaining NM and SM samples were categorized as “Low steroid hormones” as they were generally lower in all steroid concentrations compared to the other clusters.

### Principal component analysis (PCA) results

**Figure 4:**
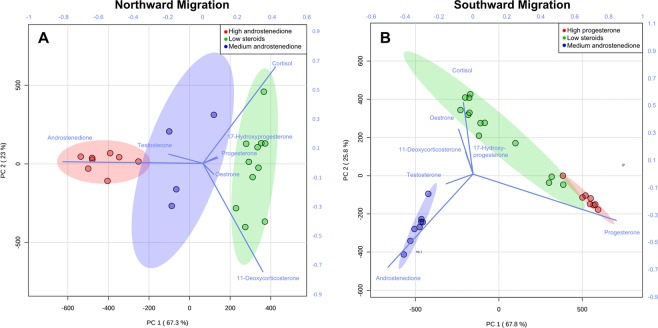
Principal component analysis biplots of (**A**) the “Northward Migration” (NM) and (**B**) “Southward Migration” (SM) sample sets. NM samples were classified as “High androstenedione”, , “Medium androstenedione” , and “Low steroid hormones”, . SM data set features samples classified as “High progesterone”, , “Medium androstenedione” , and “Low steroid hormones”, . Colored ellipses represent 95% confidence regions for each cluster.

Sample data were investigated by PCA to assess whether considering multi-hormone sample profiles validated the clusters identified by single-hormone rank. NM sample clusters were highly redundant with the first two principal components (PC1 and PC2) explaining 90.3% of the total variance (Fig. 4A). Among these, “Low steroid hormones” profiles were completely independent, while “High androstenedione” and “Medium androstenedione” clusters were not separated at 95% confidence, largely due to androstenedione being dominant in the profiles of both populations. Similarly, almost all of the variance (93.6%) in the SM data set was explained by the first two components of PCA (Fig. 4B). Here the “Medium androstenedione” cluster formed a compact cluster isolated at 95% confidence from the “Low steroid hormones” and “High progesterone” clusters, which instead were overlapping each other. Sample S16.12 (progesterone = 4.0 ng/g) appeared to be responsible for the lack of separation between the “Low steroid hormones” and “High progesterone” clusters. Repeating the analysis by re-classifying this sample into the “Low steroid hormones” cluster did not result in separation between the two clusters.

## Discussion

Near-term pregnant females of Southern Hemisphere HWs are found along the coasts of Australia between June and October during their movement from Antarctica to the breeding grounds, where parturition occurs^26,28^. Current understanding of HW female reproductive endocrinology assumes that pregnancy is characterised by a prolonged period of elevated circulating progesterone levels, which results in high levels of this hormone in blubber^12^. The present study failed to find elevated blubber progesterone concentrations (>19 ng/g from Pallin’s pregnancy model) in any of the 23 northward travelling females biopsied during a 16-day sampling campaign (26 June-11 July). A recent population study has estimated an 11% annual increase among the East Australia HW population^29^. Such an increment would approximately correspond to a pregnancy rate of 22%, or higher, considering the high rates of HW calf mortality^30^. A pregnancy rate of >22% would correspond to at least five near-term pregnant females in our sample size of 23 individuals.Whilst random sampling may have omitted some pregnant individuals, the probability of complete exclusion in a rapidly recovering population seems unlikely^29,31^.

The complete absence of females with high levels of blubber progesterone in our northward migrating cohort warranted further investigation into the dynamics of progesterone trends during mammalian gestation. A decline in circulating progesterone close to parturition appears to be common in mammal species^32–34^. Progesterone maintains uterine quiescence through pregnancy^35^. A rapid fall in progesterone is necessary to initiate contractions in humans and it has been recorded to occur in humans and in other mammals 2 to 7 days before birth^13,36^. The location of the current study (27 °S) places our whale population within 12 to 24 days travel from the typical calving grounds, extending between 21° and 15° S^37^. However, both east and west Australian HW births have been shown to occur over a wider latitudinal range than previously identified^38,39^. The recent recognition of South-East Queensland as a suitable calving ground for HWs suggests that the current study might include females rapidly approaching parturition. Thus, a fall in progesterone to prepare for imminent birth could explain the absence of high progesterone levels in any of the females represented in the NM cohort.

Compared to humans, a relatively earlier decline in circulating progesterone has been recently characterized in bottlenose dolphins and killer whales (three and twelve month before parturition, respectively)^18–20^. The decrease in progesterone is induced by the transition from ovary-synthesised progesterone to placental progesterone metabolites. Some of these progesterone metabolites, such as 5α-dihydroprogesterone have a demonstrated agonist role towards progesterone binding sites, which allows the maintenance of pregnancy in absence of progesterone^34,40^. It cannot be excluded that a similar hormonal transition occurs during HW pregnancy.

The unexpected absence of any pregnant females among the NM cohort, as ascertained from progesterone levels alone, together with the detection of a unique cluster of females with similar blubber steroid profiles, namely the NM cluster of females identified as “High androstenedione”, lead us to propose that near-term pregnant whales may be better recognized by elevated levels of androgens. A link between androgens and pregnancy has previously been observed in cetacean species. Corkeron *et al*.^4^ measured faecal concentrations of testosterone in North Atlantic right whales and found levels of this hormone in pregnant females to be higher than in all other reproductive classes, including mature males. Further, testosterone measured in the respiratory vapour of right whales showed some of the highest values detected in confirmed pregnant females^41^. In a longitudinal study on bottlenose dolphins, testosterone concentration starts to rise, regardless of foetal sex, at four months post-conception, and keeps increasing until three months before birth^23^. Similarly, in a longitudinal study on killer whales, testosterone concentration starts to increase significantly in the luteal phase at five months post-conception, to reach a maximum nine months later (the killer whale gestation period is 18 months). Killer whale serum androstenedione also followed testosterone’s trends in concentrations approximately 30-fold higher^22^. High androstenedione and testosterone levels during advanced pregnancy have been detected in other mammalian species^42^, including humans^43,44^. In humans and goats, circulating maternal androstenedione and testosterone can be converted into oestrone and oestradiol respectively, by the aromatase enzyme CYP19A1^44,45^. Thus, a similar mechanism might explain high concentrations of these two hormones in cetaceans at the final stages of gestation.

The discrimination of near-term pregnant females from the rest of the migratory cohort proposed here is based upon clustering of androstenedione concentration; however, the concentration of testosterone, progesterone and cortisol also lend support to this categorization. Although the relatively low sample number in this work prevents a more detailed statistical comparison, “High androstenedione” females, as a group, exhibited the highest progesterone (median = 0.41 ng/g) and cortisol (0.84 ng/g) concentrations among all sample clusters from the NM season. Evidence for increased maternal circulating cortisol concentration close to parturition exists for other cetaceans^4,22,46^. Since cortisol appears to increase in other species around 10 days before birth^22^, high cortisol among “High androstenedione” samples corroborates the hypothesis that these individuals might be pregnant and rapidly approaching parturition. Oestrone, 11-deoxycorticosterone and 17-hydroxyprogesterone, were present in detectable levels only in a few samples, almost exclusively belonging to the “High androstenedione” cluster, suggesting that these three compounds might also be higher in these samples than in samples from other clusters.

Assuming that near-term pregnant females have been correctly identified as the “High androstenedione” cluster, our analysis would yield an average pregnancy rate of 36% for the 2015, 2016, and 2017 NM seasons. Despite the small sample number, this proportion matches Chittleborough’s^47^ calculated average pregnancy rate for the D population (37.2%), and it is reasonably similar to the pregnancy rate extrapolated from Noad *et al*. ’s most recent population estimate (>22%)^29^. Furthermore, among our data set, “High androstenedione” samples were found with increasing probability as time advanced during the NM sampling period, in agreement with the tendency of late pregnant females to delay the departure from the feeding grounds and join the migration at a later time than other whales^28^.

By applying a previously validated progesterone threshold for the detection of pregnancy (19 ng/g)^11^, only one HW would be classified as pregnant. In Southern Hemisphere HWs, ovulation occurs between June and October, with the highest frequency of conception recorded between the end of July and September^48^. Therefore, in this study we expected to be able to detect a larger number of individuals in the early stage of pregnancy, especially in samples obtained during the SM. It is possible that a number of conceptions could have occurred later in the season, at latitudes more southerly than our sampling area. In addition, recent successful conceptions may not be recognized due to a delay between conception and changes in circulating progesterone concentration (maternal recognition of pregnancy). However, even accounting for these factors, a resulting pregnancy rate of 3.45% (one in 29 samples) during the SM at our sampling time is extremely unusual.

The pregnancy criterion suggested by Pallin *et al*.^12^ was developed based on enzyme immunoassays measurement of progesterone in blubber biopsies taken at least two and a half months after the peak of the breeding season. Since our samples were obtained during the breeding season and analysed by LC-MS/MS instead, three non-exclusive explanations could justify the absence of early-pregnant females based on Pallin’s threshold. Firstly, it is possible that other immunoreactive progesterone metabolites, indistinguishable from progesterone by the most commonly used enzyme-based immunoassays, contributed to the higher progesterone concentrations quantified in the previous studies. Secondly, the earliest stages of pregnancy may not yet be identifiable in our samples because the sampling occurred before the maternal recognition of pregnancy, with a further delay expected between circulating hormone levels and a concomitant rise in blubber levels. Lastly, a lower progesterone threshold could be indicative of the earliest stages of pregnancy.

A confounding factor in recognising early pregnancy through progesterone concentrations during the breeding season is the coexistence of ovulating females and those in the early stages of pregnancy. As elegantly shown in killer whales by Robeck *et al*.^19^, serum progesterone in conceptive cycles only starts to significantly differ from that of non-conceptive cycles after the third week post-conception. This delay could be enhanced by analysing progesterone in external blubber layers. While changes in circulating hormones have been demonstrated to be reflected within hours in dolphin blubber^49^, higher concentrations may take longer to build up^2^. This factor may be especially significant when sampling the outer few cm  of HW blubber, the total thickness of which is typically between 10 and 20 cm.

Previous studies, which measured blubber progesterone in female HWs, identified a gap of approximately one order of magnitude between the blubber progesterone levels of apparently or confirmed pregnant individuals, and those of apparently or confirmed non-pregnant ones (Table 2). The possibility of a lower progesterone threshold for attribution of early-pregnancy status was explored based on the seasonal variations of concentrations of this steroid in our samples and on the relationship between our values and previously published baselines of blubber progesterone.

**Table 2:** HW Blubber progesterone intervals (ng/g, ww) associated with reproductive status or for inferring reproductive status in the literature.

Authors and year	Validation process	Maximum value for non-pregnant individuals	Minimum value for pregnant individuals
Clark *et al*.^9^	Assumed reproductive status	1.29	21.92
Pallin *et al*.^12^	Confirmed reproductive status	4.09	54.97
Pallin *et al*.^11^	Adopted Pallin *et al*.’ s^12^ model	4.86	19.28
Riekkola *et al*.^10^	Adopted Pallin *et al*. ’s^12^ model	5.26	25.81

In our samples, the highest concentrations of progesterone (>4.04 ng/g) were found exclusively in the SM data set. If the progesterone measured was luteal in origin, then none of the females sampled during the NM had experienced ovulation for that season at the time of sampling. This does not correspond with the previously reported proportion of females ovulating before the first half of July of approximately 20%^48^. Further, the distribution of progesterone concentrations in our samples (Fig. 2B) was relatively continuous. The maximum gap in progesterone concentrations was 4.52 ng/g, within a range between 0.0651 ng/g and 21.9 ng/g. Most of the higher values of progesterone measured in this study are in the gaps reported by previous literature to distinguish non-pregnant from pregnant whales. Since a rapid increase in progesterone concentration values was observed at approximately 2 ng/g in our data set (Fig. 2B), an arbitrary pregnancy criterion threshold of 4.04 ng/g was adopted in this study.

A number of females within both the NM and SM sample sets were distributed in the “Medium androstenedione” clusters, which were categorized as such on the basis of their relatively moderately elevated androstenedione concentrations. In killer whales, circulating testosterone and androstenedione concentrations during the luteal phase are higher than at baseline^22^. Therefore, the individuals classified as “Medium androstenedione” could be approaching or experiencing ovulation, although why their progesterone levels are basal is not explained.

Lipid percentages of the blubber samples were also quantified in this study, without finding any significant trend with the measured steroid hormone concentrations, except for cortisol. This corresponds with previous findings within stranded HWs^24^ and live biopsied bottlenose dolphins^50^. This suggests that the observed hormonal changes result from a combination of possibly unrelated seasonal effects, driven by reproductive, environmental, or metabolic changes not directly related to the individual’s lipid percentage.

In conclusion, using the current validated progesterone concentration threshold for pregnancy attribution, only one of the 52 migrating female HWs analysed in this study could be classified as pregnant. Such a low pregnancy incidence is unlikely considering the current estimated rapid recovery of this whale population^29^. Analysis of steroid hormone profiles did however, identify a clear cluster of females in the NM cohort based upon androstenedione and testosterone, thus suggesting two potential additional steroid hormone biomarkers of pregnancy. Use of androgens as indicators of late-pregnancy has been reported for cetaceans and other mammals. Additionally, the distribution of blubber progesterone concentrations at low but above-basal concentrations observed in SM females suggests that a new threshold (4.0 ng/g) should be considered to allow attribution of the earliest stages of pregnancy. The inconsistencies between previously observed elevated concentrations of progesterone and our data emphasise the importance of timing of blubber biopsy across the breeding cycle of HWs for pregnancy assessment, and encourages the development of mass spectrometric methods to measure other progestin metabolites which may be elevated during HW pregnancy. The large estimated HW population (20,000–30,000 individuals)^29^ combined with the dynamics of migration makes validating assigned reproductive categories extremely difficult, even with life-history observations. Nevertheless, the hormonal profiles obtained show discrete patterns that provide an important platform for future non-invasive research on this species.

## Materials and Methods

### Samples

Humpback whales from the east coast of Australia (stock E1 as classified by the International Whaling Commission)^51^ were sampled in Moreton Bay Marine Park, North Stradbroke Island, Queensland (approximately 27°26 S, 153°34 E). Samples were collected between 26^th^ June and 17^th^ July (Julian days 178 to 198, n = 22) through 2016 and 2017, and between the 27^th^ September and the 25^th^ October (Julian days 270 to 299, n = 30) in 2015, 2016 and 2017. At our sampling latitudes, these dates in June and July coincide with the peak of frequency of encounters with HWs swimming from the polar feeding grounds to the warmer tropical waters to breed (“Northward Migration”, NM)^52^. In August, HW adults and their nursing new-born calves start to be found swimming from the breeding grounds towards Antarctica (“Southward Migration”, SM). The inversion of frequency between the two migration routes happens in August, although individuals swimming in both directions can be observed on the migratory corridor until October^52^. In this study, only one sample, S15.04 collected on September the 14^th^, during the peak of the SM season, was observed to be swimming northward and was therefore reclassified as taking part in the NM.

HW calving is known to occur during the Northward migration and HW gestation duration has been estimated at around 12 months^26^. Therefore, conceptions that occur during migration will result in the birth of a calf on arrival at the calving grounds the following year. The sampling location of the current study in relation to the population’s calving grounds on the Great Barrier Reef^37^ dictates that exclusively near-term pregnant females should be present among females sampled during the NM, while early-pregnant females can be present during both seasons (NM and SM). The Southern Hemisphere HW endures the migration in a state of fasting, which induces a rapid reduction in blubber lipids between our sampling seasons^53,54^. On the basis of these known seasonal characteristics our analyses were conducted considering NM and SM samples separately.

HW sampling was conducted under approved scientific research permits (Moreton Bay Marine Park/Permit #QS2014/CVL1397) and Animal Ethics Approval (Griffith University, Ref No: ENV/10/15/AEC). All experiments were performed in accordance with relevant guidelines and regulations. Outer blubber samples (between 1 cm and 4 cm depth from the dorsoventral region of the whale) were randomly collected from live, free-roaming HWs by remote biopsy darts (0.8 mm to 0.9 mm Ø, approximately 3 cm to 4 cm length) as previously described by Bengtson Nash *et al*.^53^. A portion (0.1–0.4 g) of blubber tissue was subsampled from each biopsy for the hormone analysis and stored at -78 °C until extraction. Female individuals were identified by DNA-sexing of the matching skin sample collected with each biopsy according to Palsbøll *et al*.^55^. Information regarding age and sexual maturity of the sampled individuals was not available in this study. Samples from sexually immature juvenile female whales were therefore expected to be among those collected. One individual (S15.55) was identified during field operations as a calf-of-the-year (i.e. less than four months old) and therefore removed from the statistical analysis. Sampled individuals were recorded as a recent mother only when they were sighted alone with a calf-of-the-year due to the lack of photoidentification records or of complete pod sexing.

### Lipid determination

Lipid percentage was calculated for each sample from the ratio of Total Lipid Extract (TLE) mass and the initial sample mass. The TLE was extracted from approximately 0.03 g of blubber using a modified Bligh and Dyer^56^ methanol–dichloromethane–water extraction as described by Ericson *et al*.^57^.

### Steroid hormone analysis

Steroid hormone analysis in the blubber biopsy was performed on an Agilent 1200 Series HPLC liquid chromatography system coupled to an AB Sciex API 4000 QTRAP hybrid triple quadrupole/linear ion trap mass spectrometer. Sample preparation, instrumental parameters, and quantification criteria have been previously validated and described in detail^24^. Briefly, blubber samples were mechanically homogenized with the addition of water and then steroids were extracted in acetonitrile using an Agilent (Santa Clara, California) Bond Elut QuEChERS EN extraction kit followed by cleanup with a QuECheERS C18 dispersive solid phase extraction (dSPE) tube (15 mL).

LC-MS/MS measurement was conducted using the methods described in Dalle Luche *et al*.^24^. Testosterone, androstenedione, progesterone and 17-hydroxyprogesterone were separated using a gradient of acetonitrile and methanol (both containing 0.1% formic acid) on a Restek (Bellefonte, Pennsylvania) Ultra Biphenyl column (5 μm, 250 mm × 4.6 mm) heated to 35 °C. The initial acetonitrile/methanol eluent composition changed from 80% methanol to 55% methanol over 30 minutes, then to 20% methanol over one minute. After holding at 20% methanol for four minutes, the methanol content increased to 80% over 0.1 minutes, and this was held for 9.9 minutes^58^. Using a separate portion of extract, oestradiol and oestrone were first derivatised with dansyl chloride and injected onto the same column with the same mobile phase solvents as above, on an initial gradient of 20% methanol held for 20 minutes, then increased to 100% methanol over one minute, held for five minutes, decreased to 20% over 0.1 minutes, and held over 9.9 minutes. Cortisone, cortisol, corticosterone, 11-deoxycortisol, and 11-deoxycorticosterone were separated in methanol and water (both containing 0.1% acetic acid) on an Agilent Eclipse Plus C18 column (5 μm × 150 mm × 21 mm). The elution gradient started with 46% methanol held for 20 minutes, then increased in a minute to 100%, held for three minutes, decreased to 46% over 0.1 minutes, and held at 46% for 9.9 minutes. The chromatographic system was interfaced to the mass spectrometer by electrospray ionization operating in positive mode. For each compound a quantitative fragment ion and a qualitative fragment ion (for identity confirmation) were measured in selected multiple reaction monitoring mode^58^.

Standard solutions of steroids in methanol at different concentrations (calibration standards; Supplementary Information Table S1) and blanks (water) were prepared simultaneously with the samples. The concentration in the sample extracts was calculated by regression of the mass-adjusted signals obtained from the calibration standards. Isotopically labelled steroid standards were used to normalize variances in extraction and injection (Supplementary Information Table S1). The limit of detection (LOD) was calculated as three times the standard deviation plus the mean of the extracted blanks for each analyte. The reporting limit (RL) was chosen as the lowest calibration standard used within the regression with a minimum signal to noise ratio of three to one. The method limits of detections and reporting limits ranged over an order of magnitude among the analysed steroid suite (Supplementary Information Table S2). Most steroids reporting limits were between 0.028 ng/g ww and 0.24 ng/g. Corticosterone, 11-deoxycortisol, and 11-deoxycorticosterone showed higher reporting limits (between 0.50 ng/g ww and 0.64 ng/g ww) due to less efficient chromatographic resolution^24^. Coefficients of determination of the calibration standard regression were all above 0.992 (Supplementary Information Table S1).

## Statistical analysis

Data were censored at the highest RL. Values below the RL were inputted with the RL and treated with non-parametric statistical tools^59^. Single-hormone comparisons between sample groups were performed by the Kruskal-Wallis rank test (χ2, chi-squared) in RStudio (Version 1.0.136). Correlations between steroid pairs were investigated by Spearman rank (r_s_, Spearman rho). Steroids detected in less than 50% of the samples were excluded by the correlation analysis. Correlations between steroid concentrations and between steroid concentrations and lipid percentages were investigated considering the whole sample set (n = 52). When a significant correlation was found between a pair of variables, the correlation was also investigated separately in the NM and SM subsets (NM, n = 23; SM, n = 29) to better understand whether this was influenced by seasonal factors. Intra-seasonal correlations among hormonal couples not correlated in the whole data set were not reported, due to the smaller sample size, and weak statistical power of the non-parametric rank comparison. To perform PCA, values below the RL were instead inputted with a random number from between 0 and the RL. Missing 17α-hydroxyprogesterone values (not acquired due to technical issues during a relatively small number of analyses) were inputted by using the k-nearest neighbour algorithm (k-NN). The profiles were then normalised by the sum of all hormones and mean-centred. The Kruskal-Wallis rank test was performed using R (Version 3.3.2)^60^ under RStudio environment (Version 1.1.463; MA, USA)^61^. R packages “dplyr“^62^ and “ggplot2”^63^ were used. Spearman rank and Principal Component Analyses (PCA) by covariance were performed in MetaboAnalyst 4.0 (Xia Lab, McGill Univeristy, Canada). A confidence level of 95% (p = 0.05) was employed throughout the statistical analyses.

## Supplementary information

Supplementary Information.

## Data Availability

The datasets generated during and/or analysed during the current study are available from the corresponding author on reasonable request.
