# Supplementary material for: Androstenedione and testosterone but not progesterone are potential biomarkers of pregnancy in Humpback Whales (Megaptera novaeangliae) approaching parturition
Source: Sci Rep. 2020 Feb 19;10:2954. doi: 10.1038/s41598-020-58933-4 (PMC7031522; doi:10.1038/s41598-020-58933-4)
Supplement: Supplementary file 1 — Supplementary Information. [file 41598_2020_58933_MOESM1_ESM.pdf]

Manuscript's supplementary information

Supplementary information to: Androstenedione and testosterone but not progesterone are potential biomarkers of pregnancy in Humpback Whales (*Megaptera novaeangliae*) approaching parturition

Greta Dalle Luche [a]*, Ashley S.P. Boggs [b], John R. Kucklick [b], Jasmin Groß [a], Darryl Hawker [c] & Susan Bengtson Nash [a]

[a] Environmental Futures Research Institute, Griffith University, Brisbane QLD 4111

[b] National Institute of Standards and Technology, Hollings Marine Laboratory, Charleston, SC 29412, USA

[c] School of Environment and Science, Griffith University, Brisbane QLD 4111

*Corresponding author: Greta Dalle Luche, greta.dalleluche@griffithuni.edu.au

List of Tables

Table S1: Calibration standard regressions. *Pg 2*

Table S2: Limits of detections (LODs) and reporting limits (RL). *Pg 4*

Table S1: Calibration standard regressions.

Analyte	Associated Standard	Determination Coefficient (R_2)	Calibration range (ng/g)		
Batch 1					
Testosterone	Testosterone-2,3,4- $^{13}C_3$	1	0.15	to	60
17-Hydroxy-progesterone	17 α -Hydroxyprogesterone-2,3,4-$^{13}C_3$	0.995	0.13	to	140
Androstenedione	4-Androstene-3,17-dione-2,3,4-$^{13}C_3$	0.999	0.011	to	12
Progesterone	Progesterone-2,3,4-$^{13}C_3$	1	0.030	to	600
Cortisone	Cortisol-9,11,12,12- d_4	0.997	0.22	to	44
Cortisol	Cortisol-9,11,12,12- d_4	0.998	0.10	to	47
Corticosterone	17 α -Hydroxyprogesterone-2,3,4-$^{13}C_3$	0.994	0.50	to	42
11-Deoxycortisol	17 α -Hydroxyprogesterone-2,3,4-$^{113}C_3$	1	0.48	to	54
11-Deoxy-corticosterone	17 α -Hydroxyprogesterone-2,3,4-$^{13}C_3$	0.999	0.64	to	41
Oestradiol	17β-Oestradiol-2,3,4-$^{13}C_3$	0.999	0.20	to	10
Oestrone	17β-Oestradiol-2,3,4-$^{13}C_3$	1	0.11	to	17
Batch 2					
Testosterone	Testosterone-2,3,4- $^{13}C_3$	0.999	0.25	to	150
17-Hydroxy-progesterone	17 α -Hydroxyprogesterone-2,3,4-$^{13}C_3$	1	0.063	to	67
Androstenedione	4-Androstene-3,17-dione-2,3,4-$^{13}C_3$	1	0.022	to	130
Progesterone	Progesterone-2,3,4-$^{13}C_3$	1	0.028	to	1100
Cortisone	Cortisol-9,11,12,12- d_4	1	0.19	to	490
Cortisol	Cortisol-9,11,12,12- d_4	0.999	0.21	to	2100
Corticosterone	17 α -Hydroxyprogesterone-2,3,4-$^{13}C_3$	0.999	0.18	to	21
11-Deoxycortisol	17 α -Hydroxyprogesterone-2,3,4-$^{113}C_3$	1	0.53	to	20
11-Deoxy-corticosterone	17 α -Hydroxyprogesterone-2,3,4-$^{13}C_3$	0.994	0.23	to	27
Oestradiol	17β-Oestradiol-2,3,4-$^{13}C_3$	0.992	0.17	to	10
Oestrone	17β-Oestradiol-2,3,4-$^{13}C_3$	0.999	0.03	to	72
Batch 3					
Testosterone	Testosterone-2,3,4- $^{13}C_3$	1.00	0.14	to	39
17-Hydroxy-progesterone	17 α -Hydroxyprogesterone-2,3,4-$^{13}C_3$	0.996	0.11	to	31
Androstenedione	4-Androstene-3,17-dione-2,3,4-$^{13}C_3$	1.00	0.028	to	100
Progesterone	Progesterone-2,3,4-$^{13}C_3$	1.00	0.07	to	31
Cortisone	Cortisol-9,11,12,12- d_4	0.999	0.10	to	53
Cortisol	Cortisol-9,11,12,12- d_4	1.00	0.18	to	97

Corticosterone	17 α -Hydroxyprogesterone-2,3,4-$^{13}C_3$	0.999	0.26	to	62
11-Deoxycortisol	17 α -Hydroxyprogesterone-2,3,4-1$^{13}C_3$	1.00	0.53	to	46
11-Deoxy-corticosterone	17 α -Hydroxyprogesterone-2,3,4-$^{13}C_3$	1.00	0.47	to	200
Oestradiol	17β-Oestradiol-2,3,4-$^{13}C_3$	0.999	0.29	to	69
Oestrone	17β-Oestradiol-2,3,4-$^{13}C_3$	0.996	0.17	to	80

Table S2: Limits of detections (LODs) and reporting limits (RL).

Steroid compound	LOD (ng/g)	RL (ng/g)
Testosterone	0.034	0.15
17-Hydroxyprogesterone	0.14	0.26
Androstenedione	0.013	0.028
Progesterone	0.027	0.065
Cortisone	0.064	0.23
Cortisol	0.19	0.24
Corticosterone	0.015	0.50
11-Deoxycortisol	0.074	0.53
11-Deoxycorticosterone	0.13	0.64
Oestradiol	0.56	0.56
Oestrone	0.11	0.17
